# The Influence of Sex, Gender, and Age on COVID-19 Data in the Piedmont Region (Northwest Italy): The Virus Prefers Men

**DOI:** 10.3390/life12050643

**Published:** 2022-04-26

**Authors:** Silvia De Francia, Alessandro Ferretti, Francesco Chiara, Sarah Allegra, Daniele Mancardi, Tiziano Giacomo Allice, Maria Grazia Milia, Gabriella Gregori, Elisa Burdino, Claudio Avanzini, Valeria Ghisetti, Alessandra Durio

**Affiliations:** 1 Department of Biological and Clinical Sciences, University of Turin, S. Luigi Gonzaga Hospital, 10043 Orbassano, Italy; 336124@edu.unito.it (F.C.); sarah.allegra@unito.it (S.A.); daniele.mancardi@unito.it (D.M.); 2 Department of Physics, University of Turin, 10125 Turin, Italy; alessandro.ferretti@unito.it; 3 Laboratory of Microbiology and Virology ASL Turin, 10149 Turin, Italy; tiziano.allice@gmail.com (T.G.A.); mariagrazia.milia@aslcittaditorino.it (M.G.M.); gabriella.gregori@aslcittaditorino.it (G.G.); elisa.burdino@aslcittaditorino.it (E.B.); claudio.avanzini@aslcittaditorino.it (C.A.); valeria.ghisetti@aslcittaditorino.it (V.G.); 4 Department of Economics and Statistics “Cognetti de Martiis”, University of Turin, 10153 Turin, Italy; alessandra.durio@unito.it

**Keywords:** sex, gender, Coronavirus infectious disease 2019, differences, tailored approach

## Abstract

Several important sex and gender differences in the clinical manifestation of diseases have been known for a long time but are still underestimated. The infectious Coronavirus 2019 disease pandemic has provided evidence of the importance of a sex and gender-based approach; it mainly affected men with worse symptomatology due to a different immune system, which is stronger in women, and to the Angiotensin-converting enzyme 2 and Transmembrane protease serine 2 roles which are differently expressed among the sexes. Additionally, women are more inclined to maintain social distance and smoke less. Analysis of data on the infectious Coronavirus 2019 disease testing from people admitted to the Amedeo di Savoia Hospital, a regional referral center for infectious diseases, has been applied to the whole of 2020 data (254,640 records). A high percentage of data in the dataset was not suitable due to a lack of information or entering errors. Among the suitable samples, records have been analyzed for positive/negative outcomes, matching records for unique subjects (N = 123,542), to evaluate individual recurrence of testing. Data are presented in age and sex-disaggregated ways. Analyses of the suitable sample also concerned the relation between testing and hospital admission motivation and symptoms. Our analysis indicated that a sex and gender-based approach is mandatory for patients and the National Health System’s sustainability.

## 1. Introduction

Important sex and gender differences are observed in the frequency, symptoms, and severity of several diseases, in addition to the response to treatments and adverse drug reactions. A sex and gender-based approach to clinical practice can significantly contribute to health promotion by improving the appropriateness of care and, therefore, providing benefits for patients and the National Health System’s sustainability. This is true also in the context of the infectious Coronavirus 2019 disease (COVID-19). What was discovered as a cluster of patients with a mysterious respiratory illness in Wuhan, China, in December 2019, was later identified as COVID-19. The pathogen of severe acute respiratory syndrome coronavirus 2 (SARS-CoV-2), a novel Beta coronavirus, was subsequently isolated as the causative disease agent [1,2,3,4,5,6,7] and on 11 March 2020, the World Health Organization (WHO) declared COVID-19 a pandemic. A growing body of evidence reveals that the male sex is a risk factor for more severe disease: globally, approximately 60% of deaths from COVID-19 are reported in men [8]. Women seem to be more protected for different reasons. Past studies have shown that sex has a considerable effect on the outcome of infection and has been associated with underlying differences in immune responses leading to physiological and anatomical differences that may influence exposure, receptor recognition, clearance, and even transmission of microorganisms. The X-linked nature of immune response proteins deeply marks the difference: women mount a stronger immune response to infections and vaccinations and outlive men [9,10,11,12,13,14,15,16,17]. In addition to the different immune responses, Angiotensin-converting enzyme 2 (ACE2), a protein involved in blood pressure regulation and the cleavage of substrates acting in different physiological processes, plays a central role in COVID-19 sex-related progression. SARS-CoV-2 utilizes the ACE2 receptor as its main entry portal and, possibly, as a route to secondary “metastatic” end-organ disease [18,19,20,21,22,23,24]. The binding of COVID-19 spike protein to ACE2 induces the ACE2 down-regulation that leads to a decrease of angiotensin (1–7) production in the lung, igniting acute respiratory failure. Estrogen, in particular 17β-estradiol (E2), the main female sex hormone, upregulates the expression of ACE2 that, accordingly, is higher in females than in males. Therefore, E2 by ACE2 overexpression in the female sex could, at least partially, account for the better outcome and the lower death rate in female COVID-19 patients. SARS-CoV-2 interfaces furthermore with the renin-angiotensin-aldosterone system (RAAS) through ACE2 and there are concerns that RAAS inhibitors may change ACE2 expression and thus COVID-19 virulence. In addition, estrogens are believed to inhibit the activity or expression of different components of the RAAS system [25]. In the end, Transmembrane protease serine 2 (TMPRSS2) leads to continuous virus entry into cells, but its expression is upregulated by androgens [26,27]. Different immune responses, the RAAS system, ACE2, and TMPRSS2 role, and hormonal status are biological sex-related differences that count markedly towards the different COVID-19 progression among the sexes [28,29,30,31,32]. Gender, profoundly understudied, counts significantly in this context [33]. It may reflect behaviors that influence exposure to microorganisms, access to healthcare, or health-seeking behaviors that can affect the infection course. Women smoke less and show more compliance to basic rules of social distancing. Women use facial masks accurately and are more skilled with personal hygiene. Understanding these factors will not only help to gain a better knowledge of COVID-19 pathogenesis but will also guide the design of effective strategies for sex and gender-based personalized medicine. The supranational organization Global Health 50:50 [34] requested participating nations to report the sex and gender-disaggregated clinical data related to COVID-19 incidence and its mortality. However, to date, most clinical specialists continue to analyze data without any categorization. Our analysis highlights this point, recognizing a central role in categorizing data according to sex and gender differences.

The aim of this work was to analyze data on COVID-19 testing in the Piedmont region, northwest Italy, for people admitted to the Amedeo di Savoia Hospital, a regional referral center for infectious diseases. Data are referred to for the whole of 2020.

## 2. Materials and Methods

### 2.1. COVID-19 Testing

Analysis was performed on the COVID-19 testing dataset obtained from the database used by the Microbiology and Virology Laboratory of the Amedeo di Savoia Hospital, a regional reference center for infectious diseases. The original dataset constituted 254,640 records and 10 analytical variables referred for the whole of 2020 (from 1 January to 31 December). The data analyzed were not attributable to identity data (name and surname); each record was immediately encoded with a specific identification code. The statistical software used for analysis was R (R Core Team 2017) and its text-mining (TM) packages [35,36,37,38]. The use of these packages to perform the statistical analysis is based on the evidence that health care professionals produce abundant textual information in their daily clinical practice, stored in many different sources. The extraction of insights from all the gathered information, mainly unstructured and lacking normalization, is one of the major challenges in computational medicine. In this respect, TM assembles different techniques to derive valuable insights from unstructured textual data, so it becomes especially relevant in medical analyses. The work of cleaning and data editing on the COVID-19 testing dataset was carried out with handmade checks scrolling through every single record. This allowed us to (1) define new variables by cross-checking some information contained in the date of birth, tax code, and date of the test, and (2) allowed us to enhance some missing values to create a unique subject identifier. The dataset resulting from all editing operations was therefore made up of 251,657 records and 19 variables. We identified the frequency of regional distribution for patients tested for COVID-19 origin and analyzed some information about the test performed: the type of test done; the execution date, considered on four different year periods (I: February-May, II: June-August, III: September-October, and IV: November-December) decided retrospectively from evaluation of pandemic trend; and the positivity and negativity percentage rate related to the different defined periods.

### 2.2. Unique Subjects

To better understand the characteristics of the subjects who have undergone at least one COVID-19 test during 2020, unique subjects have been labeled, identifying them through the code-specific tax key. Data have been presented in a sex and age-disaggregated way, considering different age classes. The distribution of tests for unique subjects during 2020 has been analyzed and matched with the test result, selecting for each unique subject the first positive test obtained, if present, and for subjects never testing positive, their first result is listed in the dataset.

### 2.3. Epidemiological Criterion

Among the variables of the original dataset, there was that relating to the epidemiological criterion, an open and non-mandatory field. The operator could fill it out by writing text with the contents he/she considered most significant. This variable potentially contained lots of information about subjects tested for COVID-19, but this information was not directly analyzable with common statistical tools. Using TM, it was possible to maximize the information obtainable from this field. The nature of this field entailed a number of obstacles to the use of recorded information; first of all, not being a mandatory field, many records did not contain any text. Furthermore, being a free field, the contained information was dependent on the operator who compiled the record corresponding to the COVID-19 test done. What appeared in the text of the criterion field could be very detailed, including information on health, such as symptoms or concomitant pathologies, of the subject tested and/or the reasons for having done the test, but it could also be used only as a field for notes, containing telephone numbers, email contacts, or personal names of the attending physician or reference contact. Being a text field, it was edited by the operator and therefore subjected to typos and grammatical errors, the use of acronyms, abbreviations, and synonyms, generating significant confusion. To manage the information contained in the epidemiological criterion present in the dataset, we used TM techniques which allowed us to transform texts into structured data. At first, we had to clean the dataset, removing empty records for epidemiological criteria. The resulting dataset was therefore made up of 196,970 records. The corpus of documents that we analyzed consisted of 196,970 texts (the epidemiological criteria). We then proceeded to standardize the texts of each document through different operations: the conversion of all characters to uppercase or lowercase; the elimination of special characters such as punctuation, multiple spacing, symbols, or numbers; the elimination of all stop-words, the common language words that did not add meaning to the sentence content, such as articles, pronouns, adverbs; and the transformation of words into lemmas, that is the transformation of a word into its canonical form. We turned each document into a word vector (unigram) and created the bag of words (BofW) matrix of 196,970 rows, one for each criterion, and 23,022 columns, one for each different word contained throughout the corpus. In the cells of the BofW matrix, there was the number of times that the column word was present in the row criterion. The 23,022 words identified in this first phase form the dictionary of the document’s corpus corresponding to the criteria. For the problems described above, these 23,022 words did not identify as many different concepts, many were synonymous, others were misspelled, and still, others were abbreviations. For example, in the resulting dictionary, we found 55 different ways in which the term “COVID” was spelled. There is no statistical method that can automatically identify these 55 words and reduce them all to the “COVID” unigram, it was, therefore, necessary to correct the dictionary manually by reading the 23,022 words and indicating their possible correspondence to its correction. Subsequently, we proceeded to correct the data with a string replacement algorithm. This correction operation has been repeated twice in trying to unify in a single term the plural, singular, male, and female forms. For example, different Italian words meaning “sanitary” (sanitari, sanitario, and sanitaria) have all been reduced to one term “sanitariao”. In the end, we reduced the dictionary to 9683 unigrams.

### 2.4. Definition of Subject and Symptoms Categories

**Figure 1:**
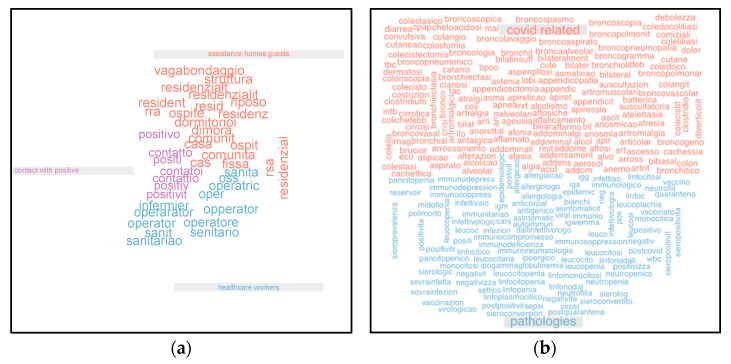
(**a**) The clusters of terms defining different subjects’ categories that have undergone the COVID-19 test. (**b**) The clusters of terms defining different symptom categories of subjects that have undergone the COVID-19 test.

First, looking at the entire dictionary, we defined three subsets of terms corresponding to a separate given category of subjects. These three subsets are defined by the terms written with the same color in Figure 1a. The subset of the terms in blue identifies the category of healthcare workers, the terms in purple identify the category of those who had contact with a positive subject, and the subset in red is related to the category of assistance home guests. Afterward, if the criterion contained at least one of the terms of a subset, the dataset record was classified as a test mate to a subject of the corresponding category. In this way, we added to the original dataset three new dichotomous variables, one for each category, the result of structuring the information contained in the textual description of the criterion. Going into more detail, we identified unigrams related to the definition of three different subject categories: healthcare workers; assistance home guests; and contacts with a positive subject. We then proceeded to subject categories analysis, studying them separately.

A comparative study was carried out between categories and the presence of symptoms. Coming back to the entire dictionary, we defined two subsets of terms, each corresponding to a given macro category of symptoms. These two subsets were defined by the terms written with the same color in Figure 1b. The subset of the 597 terms in blue identifies the category of other different pathologies (P), while the 773 terms in red identify the category of COVID-19 related symptoms (S). Afterward, if the criterion contained at least one of the terms of a subset, the dataset record was classified as a test mate to a subject of the corresponding category. In this way, we added to the original dataset two new dichotomous variables, one for each symptom category. General analysis of symptoms, furthermore, was referred in a sex, age, and test outcome-disaggregated way.

## 3. Results

We showed the main results of the analyses performed. What is not included in the following figures and tables can be found in the Supplementary Paper Material. We did not report the *p*-values of statistical analyses performed because, with such high numbers, the difference between subpopulations considered was always statistically significant.

### 3.1. COVID-19 Testing

**Figure 2:**
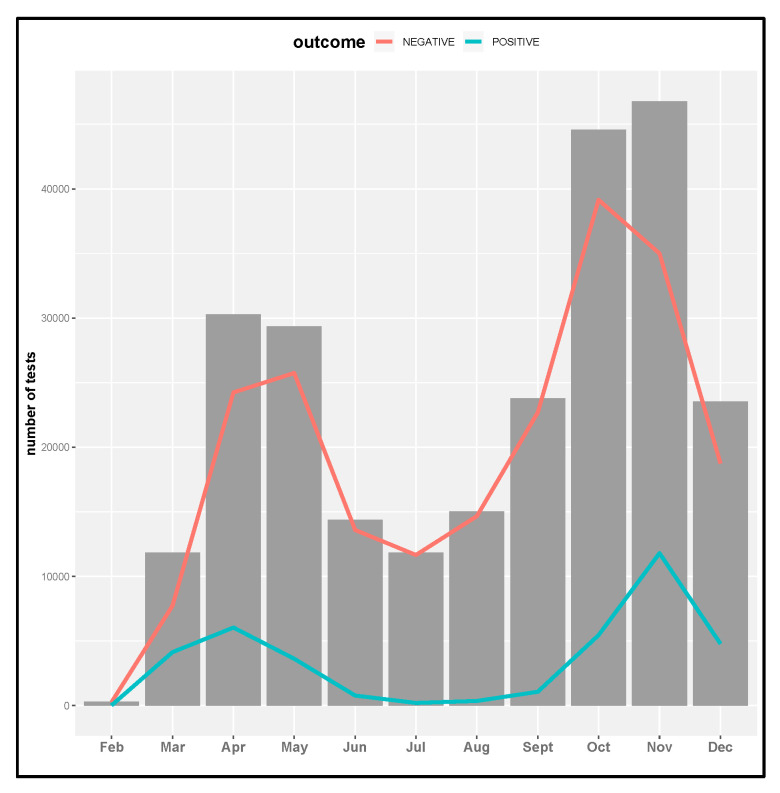
The monthly time series of COVID-19 tests.

COVID-19 tests were made on patients coming from 18 different local health districts: 80% from the Turin city district and 20% attributable to patients coming from Turin province local health districts. Different types of tests were performed: for 18% of tests there was no information, 69.5% were nasal and nasopharyngeal tests, 9.3% were nasopharyngeal, 1.8% were nasal tests, and the residual percentage referred to a pharyngeal, bronchoaspirate, or bronchoalveolar washing or salivary tests. The date of test execution was analyzed in terms of frequency referred to the whole of 2020; the bar graph in Figure 2 shows the monthly time series of tests. In the same figure, the red line shows the monthly time series of tests with a negative outcome and the red line of tests with a positive outcome. There were two peaks in the incidence of infection coinciding, respectively, with the spring (April–May) and autumn months (October–November).

With respect to the defined four periods of the year, we stated that in the first period, (I: February–May) 28.5% of tests were performed; in the second period (II: June–August), 16.4% of tests were performed; in the third period (III: September–October), 27.2% of tests were performed; and in the fourth period (IV: November–December), 27.9% of tests were performed. Positive and negative percentage rates related to different periods were analyzed: in the first period, 71,765 COVID-19 tests were done and 13,812 were positive (19.2%); in the second period, 41,222 COVID-19 tests were done and 1322 were positive (3.2%); in the third period, 68,355 COVID-19 tests were done and 6524 were positive (9.5%); in the fourth period, 70,315 COVID-19 tests were done and 16,582 were positive (23.6%).

### 3.2. Unique Subjects

A total of 123,542 unique subjects have been identified: 54.7% were female subjects and 45.3% were male. As distribution in the four periods, we observed that 31.9% of unique subjects were tested in the first period, 19.0% in the second, 26.6% in the third, and 22.4% in the fourth. A total of 80.6% of unique subjects (N = 99,595) were tested for COVID-19 detection in only one of the four periods, while 2% (N = 2501) were tested in each period, and 6% (N = 7413) were tested in three out of the four periods. Sex and age-disaggregated data analysis has been done. In Table 1, we provide the main age distribution parameters (minimum, maximum, mean, median, and quartiles) by sex and period. On average, a subject repeated the test during the year two times, furthermore, 90.5% of the subjects repeated the test no more than four times. However, 11.7% of unique female subjects were tested for COVID-19, more than 4 times that of men, 6.8%.

**Figure 3:**
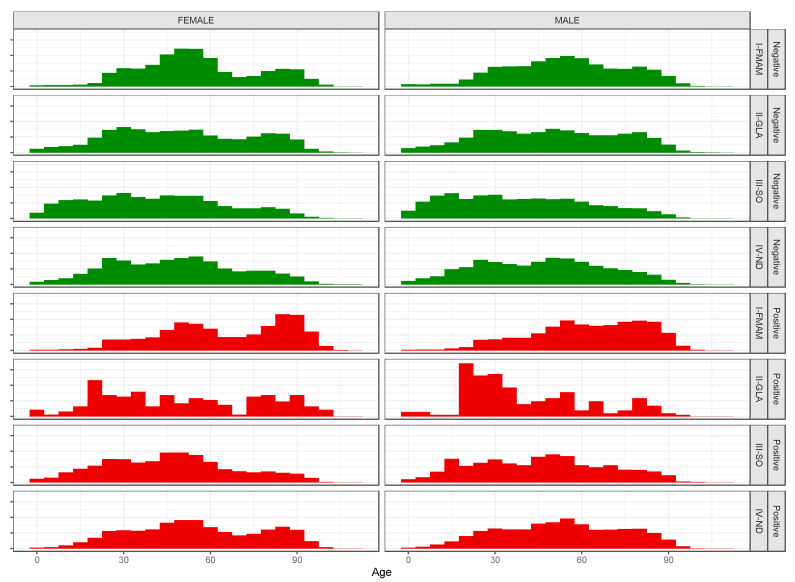
Age distributions by sex (left column = Female, right column = Male), outcome test type (green = Negative, red = Positive), and periods (two pairs of four rows).

The number of women was higher than the number of men in each period-related percentage. The median age was, in general, a similar value among sexes (49 for males and 50 for females), but considering the age distribution in the four periods, it was shown that for both males and females, the average and median age was inversely correlated to the period, decreasing over time during 2020. Analysis of unique subjects for test results has also been done, selecting the first positive test obtained, if present. We observed 97,839 subjects who were never positive: 70,788 with a single test and 27,051 with multiple negative tests. We chose the first result listed in the dataset. Furthermore, we observed 25,703 subjects with at least one positive test: 9933 with only one positive test and 15,770 with multiple tests, positive and negative. For them, we chose their first positive test listed in the dataset. Then, we analyzed tests results in a sex and age-disaggregated way for each period. Regarding the whole year, 79.19% of the subjects had a negative result and 20.81% had a positive result. Looking at the distribution of positive tests in the periods, 34.9% were in period I, 1.5% in II, 19.6% in III, and 43.9% in IV. Considering the male percentage with positive tests, we observed growth from 33.6% (I) to 44.2% (IV), with an increase of 10.60%; for the positive female percentage, the increase was 7.7% (from 36.1% in period I to 43.8% in period IV). The average and median age for both positive and negative subjects, men and women, inversely correlated to period, decreasing over time during 2020. Going into more detail, considering the age distributions by period, sex, and test type outcome in Figure 3, we observed that in the I period, ages for positive tests (see red histograms) were higher for both females (left column) and males (right column), in the II, they are lower, starting from the age class of 18–25 years; in the III and IV periods, they are in the middle ages. Furthermore, focusing on the cumulative percentage data for positive subjects aged less than 25 years, we observed that only 4% of them were located in the I period, in the second they are 26.1%, in the third 21.1%, and in the fourth 11.1%.

### 3.3. Epidemiological Criterion

**Figure 4:**
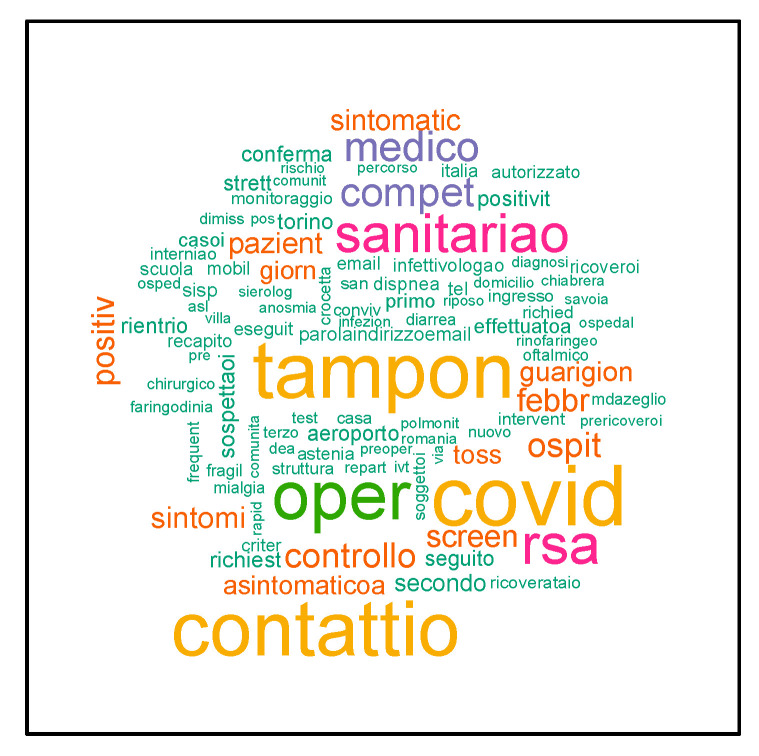
The most common words in the corpus of epidemiological criteria.

Using TM, we maximized the information obtainable from the epidemiological criterion field; we transformed texts into structured data through TM techniques. After dataset cleaning from empty spaces, the resulting dataset was made up of 196,970 records (21.7% of records were eliminated). After dataset standardization of texts in epidemiological criterion space and turning each criterion into a word vector (unigram), we created a BofW matrix composed of 196,970 rows and 23,022 columns. The 23,022 words identified have been corrected manually, indicating their possible correspondence to its correction. After being replaced with a string algorithm, we obtained 9683 unigrams. The first 100 most recurring in the criteria correspond to the words in the word cloud of Figure 4, where the font size representing the word depends on how often this word was used in the criteria, i.e., words written in large are present in many criteria.

### 3.4. Analysis of Subjects’ Categories

From the analysis of different subject categories in the 9683 unigrams dictionary identified from the TM analysis of epidemiological criterion, we obtained distinctive results. At first, a comparative study was carried out between different categories and test results obtained during 2020 and the four different year periods. For the three different subject categories identified (healthcare workers, assistance home guests, and contact with a positive subject) we obtained the following results.

Healthcare workers: 43,020 records throughout the period, 93.6% negative and 6.4% positive. The healthcare workers’ proportion of positive tests in the different periods swung, high in the first (31.6%) and the fourth (23.5%) and lowest in the second period (8.4%). Comparing the healthcare workers with the other subject categories (see the top lines of Table 2), we observed that the percentage of positive tests for others was always higher (17.0% positive) than the percentage of positive healthcare workers (6.4%) and this happened for each period (I: 23.6% for others vs. 8.6% for healthcare workers; II: 3.6% for others vs. 1.2% for healthcare workers; III: 10.9% for others vs. 3.0% for healthcare workers; and IV: 32.2% for others vs. 13.3% for healthcare workers).

In order to study the category of the assistance home guests, we removed from the dataset all the records relating to healthcare workers. The following results refer, therefore, to 153,950 records related to subjects who are not healthcare workers. Assistance home guests had a total of 26,715 records throughout the period, 83.0% negative and 17.0% positive. The proportion of assistance home guests tested for COVID-19 in the different periods was decreasing, high in the first (23.3%), going down more and more until the last period (11.9%). Looking at the positive percentage compared with others (see central lines of Table 2), we observed that in the first and second periods, the proportion of positives among the assistance home guests was higher, while in the last two periods, the relationship was reversed (I: 22.7% for others vs. 26.7% for assistance home guests; II: 3.3% for others vs. 5.1% for assistance home guests; III: 11.9% for others vs. 5.1% for assistance home guests; and IV: 33.1% for others vs. 25.6% for assistance home guests).

Contact with a positive subject had 33,603 records throughout 2020. These subjects were tested for COVID-19 because they had contact with a positive subject. Positive tests resulting from this category were 23.7%, and 76.3% obtained negative results. The contact with a positive subject proportion tested for COVID-19 in the four different periods was very high in the last period (41.9%), very low in the second period (7.4%), and started to rise in the third (16.9%). Looking at the positive percentage compared with others who are not healthcare workers (see the bottom lines of Table 2), we observed that in the first, second, and third periods, the proportion of positives among contact with a positive subject category was higher, while in the last period the relationship was reversed (I: 22.6% for others vs. 26.6% for contacts with a positive subject; II: 3.5% for others vs. 5.2% for contacts with a positive subject; III: 10.2% for others vs. 14.7% for contacts with a positive subject; and IV: 33.2% for others vs. 30.8% for contacts with a positive subject).

### 3.5. Analysis of Symptoms

Considering the analysis of the symptoms in general, we observed that for the records having one of the words classified as symptoms S in the criterion were 14.6% of the total, while the P category percentage was lower (6.2%). The percentage of positives for those who had symptoms S was greater than that of positives who did not have symptoms (23.0% vs. 13.3%), while for the type P symptomatology, related to other pathologies, the situation was reversed. Among those who did not have symptoms, the positives were 15.1% while among those who had symptoms the positives were 9.3%.

We decided to analyze in detail the category of S symptoms. Looking at the data in different 2020 periods, we noticed that in the fourth period, the percentage of positives was greater among those who did not have symptoms (31.3% vs. 25.4%), which may be due to missing filled in data in the epidemiological criterion field, due to the huge stress at the end of 2020.

**Figure 5:**
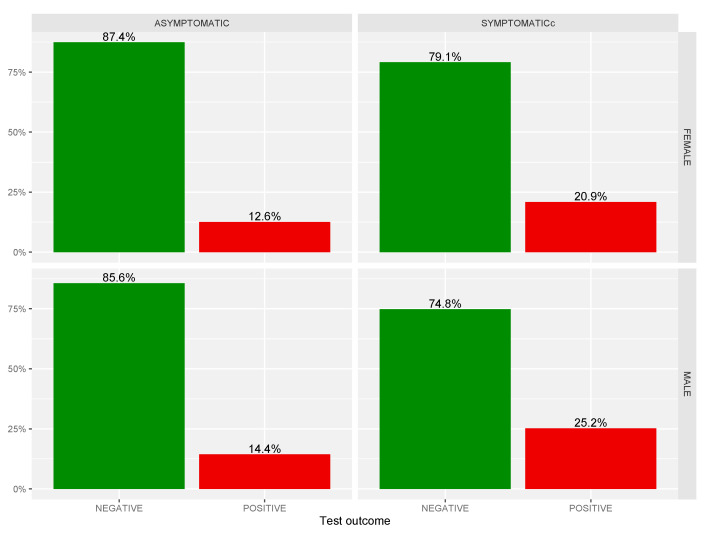
Test outcomes for COVID-19 related symptoms in a sex-disaggregated way.

This trend was also observed in the second period (3.6% vs. 1.1%) but not in the first and in the third periods where the percentage of positives was greater among those who had symptoms (I: 30.3% vs. 15.9%; III: 20.9% vs. 7.8%). In the fourth period, many tests were for positive contact so there were many positive subjects who did not yet develop COVID-19 symptoms. For these reasons, we decided to focus our analysis on symptoms not in relation to different 2020 periods. General analysis of COVID-19 related symptoms was referred to in a sex, age, and outcome test-disaggregated way. For sex, we observed that of 196,970 tests, 41.1% were done on males and 58.9% on females. The positives among males were 16.2% and 13.6% among females. We noted that among males, the proportion of those who had symptoms was 17.3%, while among females it was 12.8%. For both sexes, we observed that the proportion of positives among those who had symptoms was higher than that of the positives who had no symptoms. The difference for males was 10.9% (25.2–14.4%) while for females it was 8.3% (20.9–12.6%). Comparing males and females with symptoms, 25.2% of males were positive while 20.9% of females were positive (see Figure 5).

Regarding age, we observed that it was a risk factor for more severe disease; positive asymptomatic were younger than those positive with COVID-19 related symptoms (42.5% of symptomatic positives were aged less than or equal to 55 years while 53.1% of asymptomatic positives were aged less than or equal to 55 years). Looking at the data disaggregated by sex in Table 3, we observed that for males, 40.9% of symptomatic positives were aged less than or equal to 55 years while 56.2% of asymptomatic positives were aged less than or equal to 55 years, and for females, 44.3% of symptomatic positives were aged less than or equal to 55 years while 50.7% of asymptomatic positives were aged less than or equal to 55 years. Additionally, the male sex was a risk factor for more severe disease in terms of symptoms.

Regarding the presence of comorbidities, 5700 records were found to be related to this factor among subjects affected by COVID-19-related symptoms. Analyzing data in a sex-disaggregated way, we observed no differences; fewer positive men and women were affected by concomitant pathologies compared to positive subjects not affected by other pathologies.

A comparative study was carried out between categories and the presence of symptoms. For symptoms, we identified 1370 words divided into two macro-categories (S: COVID-19 related; P: other different pathologies).

A total of 3.0% of healthcare workers had COVID-19 related symptoms considering each period in all of 2020 (vs. 17.8% for others). The percentage of others who had symptoms was always higher than the percentage of healthcare workers with symptoms (I: 28.7% for others vs. 4.1% for healthcare workers; II: 13.7% for others vs. 2.7% for healthcare workers; III: 12.3% for others vs. 1.9% for healthcare workers; and IV: 12.8% for others vs. 0.9% for healthcare workers). This data may be explained by the need for healthcare workers to be tested for COVID-19 for monitoring purposes.

The 3.7% of assistance home guests who had COVID-19 related symptoms, considering all the 2020 and looking only at those we observed, was higher compared to others’ percentage (40% vs. 22.4%).

The 18.2% of subjects who had contact with a positive subject had COVID-19 related symptoms, considering all of 2020 and looking only at those we observed, was higher compared to others’ percentage (29.3% vs. 21.3%). Finally, the percentage of others who had symptoms was always higher than the percentage of those who had contact with a positive subject with symptoms, except in the last period (I: 29.8% for others vs. 26.6% for contact with a positive subject; II: 14.5% for others vs. 3.3% for contact with a positive subject; III: 13.6% for others vs. 5.0% for contact with a positive subject; and IV: 6.9% for others vs. 21.0% for contact with a positive subject).

## 4. Discussion

From the first 2019 reports from China, a sex imbalance with regard to detected cases and case fatality rate of COVID-19 was observed. As the disease spread across multiple continents, the *Global Health 50/50 research initiative* presented an impressive overview of sex-disaggregated data from countries worldwide, clearly demonstrating similar numbers of cases in women and men, but an increased case-fatality in men [34]. The sex disparity of COVID-19–related morbidity and mortality is likely explained by a combination of biological sex differences, such as hormonal and genetic (immune response, RAAS system, and the ACE2 and TMPRSS2 role), and gender-specific factors, such as differential behaviors and activities by social and cultural or traditional roles. Men are more likely to engage in poor health behaviors (smoking and alcohol consumption) and have higher age-adjusted rates of pre-existing co-morbidities associated with poor COVID-19 prognosis, including hypertension, cardiovascular disease, and chronic obstructive pulmonary disease [39].

Nevertheless, sex-disaggregated data are still not provided by all countries, and neither the interaction of sex and age is usually visible in the public databases.

We analyzed data on COVID-19 testing in the Piedmont region, northwest Italy, for people admitted to the Amedeo di Savoia Hospital, a regional referral center for infectious diseases, during 2020. Our undertaking was conducted to better understand the characteristics of the subjects who have undergone at least one COVID-19 test during 2020. During the time of tailored medicine, our intent was to study the variables of the whole population registered in the database used by the Microbiology and Virology Laboratory of the Amedeo di Savoia Hospital for COVID-19 testing.

From the analysis of the execution date of the test in four different year periods (I: February–May, II: June–August, III: September–October, and IV: November–December) decided retrospectively to evaluate the pandemic trend, we observed that in periods I, III, and IV the number of tests performed was almost the same, while in II (summer) it was lower. For the whole of 2020, we observed that the percentage of positive COVID-19 tests was 15.2%. In the IV period, we observed a higher positivity rate (23.6%) than the rate observed in the I period (19.2%). Questions still needed to be addressed are: who was tested in different periods? Certainly, in the first period, the number of COVID-19 tests made was very poor. Analysis of unique subjects showed the presence of a higher female percentage. This greater value may be due to the fact that throughout 2020, tests were made almost exclusively for health workers, mostly composed of female subjects, and in nursing homes, mainly inhabited by women, due to the greater presence of women in the older population segment. Analysis of test distribution for unique subjects during 2020 also showed that 8% of them had been monitored for COVID-19 detection in three or four different periods (N = 9914); this could be related to the need to control infection in affected patients or to the work-related monitoring for health personnel. Analyzing data in a sex and age-disaggregated way, we observed major tests done on the female unique subject. The number of women was higher than the number of men in each period-related percentage which is realistic if we think about women’s major involvement in the healthcare workload. Looking at the age distribution in the four periods, we observed a decrease over time during 2020 for both sexes, verifying a potential and progressive expansion of the tests to younger subjects. This may be due to the involvement of different categories of people not specifically requested to be tested for COVID-19 for professional reasons. The major female connection in COVID-19 tests, due to their major involvement in the healthcare workload, is also confirmed by the analysis of test repetition: in all periods, 11.7% of unique female subjects were tested for COVID-19 detection more than four times, vs. 6.8% of men. Analysis of test results further described the population through the positive and negative rates of infection of the population involved. In spite of the greater number of tests conducted on women, the percentage of positives among sexes was similar and we observed growth over time during 2020 from 33.6% (I) to 44.2% (IV) for men, with an increase of 10.6%, while an increase of 7.7% (from 36.1% in period I to 43.8% in period IV) was observed for women in terms of positivity rate. The observation about age distribution was confirmed also considering positive and negative subjects: the age distribution decreases, for both men and women, over time during 2020, possibly due to the admission to tests, as already mentioned, for younger subjects, i.e., students. Going into more detail, focusing on cumulative percentage data for positive subjects aged less than 25 years, we observed that fewer percentages were located in the I and IV periods, corresponding to the distance learning periods for students during the year. Analysis of epidemiological criteria was difficult because it was characterized by little standardized, very variable, and error-rich data, due to the open and non-mandatory nature of this field. The operator could fill it out by writing text with the contents he/she considered most significant, but this variable was potentially full of information that is not directly analyzable with common statistical tools. Through TM techniques, it was possible to transform texts into structured data. This underlined the importance of specific training for operators in entering additional information on data collection platforms. A uniform and codified system would have allowed us to perform an easier analysis, as well as allow more results to be obtained. However, TM techniques are not within everyone’s reach. The analysis of the epidemiological criterion through TM was conducted with different purposes. In the reduced 9683 unigrams dictionary, analysis of different subject categories identified and matched with test results and the presence of symptoms, gave us some information about the trend of the vaccination campaign, which started at the end of 2020, indicating that the need for testing decreased. Comparing, furthermore, the percentage of symptoms present, which were always higher for others than for healthcare workers, gave us the information that healthcare workers had done a large amount of testing for monitoring. The data referring to assistance home guests brought us back to the terrible period we all lived in the management of the COVID-19 pandemic. Isolation of older patients and incorrect choices led to high positivity numbers. Assistance homes were, for improvised management, especially at the beginning of pandemic, outbreaks; if an old man had a fever, he had twice the probability of being positive as one who did not live there. Separately studying the category of those who had contact with a positive subject allowed us to trace a trend for the whole of the 2020 track, confirming the usefulness of lockdowns. Additionally, the relation of testing to symptoms could furthermore allow us to better underline the potential screening role of COVID-19 tests. As the first limit of this study, we should admit the nature of the field “epidemiological criterion”, which we imagine is little or badly filled out by operators in times of great stress. In the end, and as a central point of the huge work conducted, interesting information on the disease outcome that we obtained from a general analysis of COVID-19 symptoms referred in a sex, age, and outcome test-disaggregated way, showed that male sex and older age were risk factors for more severe disease.

A further limitation of this study is the absence of correlation with mortality data, but this variable was not present in the original database since the census of the deceased was not among the objectives of the regional referral center for infectious diseases through the platform. Looking at the data collected for each Italian region by the National Istituto Superiore di Sanità referring to 2020, we observed a greater lethality of the male sex which is consistent with our observations [40].

## 5. Conclusions

Gender medicine does not exist [41]. What should definitively exist is a medical approach tailored to the variables and characteristics of each subject needing clinical assistance. We believe that observations obtainable from studies like ours could have a central role in a good preventive health policy. Statistical models like ours could be applied in general for human diseases, giving the opportunity to better understand the mechanisms underlying pathologies in the interest of the whole community. Regarding COVID-19, our study shows important implications for public health policy, illustrating the right direction for government policies for future pandemics and emphasizing intervention for those who need it most.

## Figures and Tables

**Table 1:** Age distribution parameter by period and sex for unique subjects.

			Age					
	Period	N°	Min.	1st Qu.	Median	Mean	3rd Qu.	Max.
**All Subjects** (123,542)	I-FMAM	39,517	0	43	55	56.86	74	108
	II-JJA	23,490	0	30	49	49.53	69	106
	III-SO	32,840	0	22	40	41.07	58	104
	IV-ND	27,695	0	32	50	49.81	66	109
**Male** (55,974)	I-FMAM	15,510	0	42	56	56.25	73	108
	II-JJA	11,387	0	30	49	48.99	68	106
	III-SO	16,110	0	20	39	40.05	58	102
	IV-ND	12,967	0	31	49	48.74	65	101
**Female** (67,568)	I-FMAM	24,007	0	43	55	57.26	75	107
	II-JJA	12,103	0	31	48	50.03	71	103
	III-SO	16,730	0	24	41	42.06	58	104
	IV-ND	14,728	0	33	50	50.75	67	109

**Table 2:** Test outcome by subjects’ category in each year.

Category	Period	Category Belonging	% Negative	% Positive	Total Records
**Healthcare Workers**	I-FMAM	Yes	91.3	8.73	22,216
		No	76.4	23.64	48,170
	II-JJA	Yes	98.8	1.20	4568
		No	96.4	3.63	34,019
	III-SO	Yes	96.9	3.04	13,744
		No	89.0	10.96	44,735
	IV-ND	Yes	86.7	13.32	2492
		No	67.8	32.19	27,026
**Assistance Home Guests**	I-FMAM	Yes	73.3	26.70	11,239
		No	77.3	22.71	36,931
	II-JJA	Yes	94.9	5.09	6188
		No	96.7	3.31	27,831
	III-SO	Yes	94.9	5.11	6067
		No	88.1	11.88	38,668
	IV-ND	Yes	74.4	25.64	3221
		No	66.9	33.08	23,805
**Contact with a** **Positive Subject**	I-FMAM	Yes	73.4	26.60	12,207
		No	77.4	22.64	35,963
	II-JJA	Yes	94.8	5.19	2525
		No	96.5	3.51	31,494
	III-SO	Yes	85.3	14.74	7549
		No	89.8	10.19	37,186
	IV-ND	Yes	69.2	30.83	11,322
		No	66.8	33.18	15,704

**Table 3:** Test outcome by sex and age for asymptomatic and symptomatic COVID-19 related subjects.

	Sex	Age	Negative	Positive
**Symptomatic**	Male	From 0 to 55	5209 (49.8%)	1442 (40.9%)
		Greater than 55	5242 (50.2%)	2085 (59.1%)
	Female	From 0 to 55	6068 (51.8%)	1371 (55.7%)
		Greater than 55	5642 (48.2%)	1724 (55.7%)
**Asymptomatic**	Male	From 0 to 55	34,791 (60.6%)	5406 (56.2%)
		Greater than 55	22,609 (39.4%)	4214 (43.8%)
	Female	From 0 to 55	53,249 (60.2%)	6453 (70.7%)
		Greater than 55	35,189 (39.8%)	6276 (49.3%)

## Data Availability

All codes and routines used for data analysis are available to readers upon request to alessandra.durio@unito.it. The original database is not public and is property of the Piedmont Region.

## Supplementary Materials

The following supporting information can be downloaded at: https://www.mdpi.com/article/10.3390/life12050643/s1.
