# Supplementary material for: The Influence of Sex, Gender, and Age on COVID-19 Data in the Piedmont Region (Northwest Italy): The Virus Prefers Men
Source: Life (Basel). 2022 Apr 26;12(5):643. doi: 10.3390/life12050643 (PMC9148110; doi:10.3390/life12050643)
Supplement: Supplementary file 1 [file life-12-00643-s001.zip › life-1684543-supplementary.pdf]

1-COVID-19 testing

tab1.1	All dataset records	
Health districts	num	%
ASL CITTA' DI TORINO	202811	80.59%
ASLTO4	20657	8.21%
ASLTO3	9830	3.91%
ASLTO5	5354	2.13%
CITTA' DELLA SALUTE	4122	1.64%
AO MAURIZIANO	3881	1.54%
AOUSANLUIGI	895	0.36%
ASLAL	845	0.34%
ASLCN1	811	0.32%
ASLVC	606	0.24%
ASLAT	580	0.23%
ASLCN2	513	0.20%
ASLNOVARA	285	0.11%
ASLBI	186	0.07%
ASLVCO	113	0.04%
AO CUNEO	75	0.03%
AO ALESSANDRIA	69	0.03%
AOU MAGGIORE NOVARA	19	0.01%
ND	4	0.00%
CAMPIONAMENTI ESTERN	1	0.00%
	251657	

tab1.2	All dataset records			
	Test Outcome			
Periods	NEGATIVE	POSITIVE	tot. period	%tot. period
I-FMAM	57953	13812	71765	28.52%
II-JJA	39900	1322	41222	16.38%
III-SO	61831	6524	68355	27.16%
IV-ND	53733	16582	70315	27.94%
**tot.**	213417	38240	251657	100.00%
**%tot. Outcome**	84.80%	15.20%	100.00%	

tab1.3	All dataset records		
	Test Outcome		
	NEGATIVE	POSITIVE	tot period
I-FMAM	57953	13812	71765
	80.75%	19.25%	100%
II-JJA	39900	1322	41222
	96.79%	3.21%	100%
III-SO	61831	6524	68355
	90.46%	9.54%	100%
IV-ND	53733	16582	70315
	76.42%	23.58%	100%

2-Unique Subjects_1

tab2.1 **Unique subjects (Sex)**

sex	#	%
**F**	67568	54.69%
**M**	55974	45.31%
**tot**	123542	100.00%

tab 2.3 **Unique subjects (Sex and periods)**

	**Sex**			
**Period**	**F**	**M**	tot	%tot
I-FMAM	24007	15510	39517	31.99%
II-JJA	12103	11387	23490	19.01%
III-SO	16730	16110	32840	26.58%
IV-ND	14728	12967	27695	22.42%
tot	67568	55974	**123542**	100.00%
%tot	54.69%	45.31%		

tab2.4 **Unique subjects (How many periods in which the subjects were tested)**

	**Number of periods**				
	1	2	3	4	
# subjects	99595	14033	7413	2501	**123542**
%	80.62%	11.36%	6.00%	2.02%	

tab2.5 **Unique subjects (Number of time a subjects has taken the test in the year)**

	**all**			**Male**			**Female**		
**# of time**	**#**	**%**	**Cum%**	**#**	**%**	**Cum%**	**#**	**%**	**Cum%**
**1**	80721	65.34%	65.34%	38257	68.35%	68.35%	42464	62.85%	62.85%
**2**	18321	14.83%	80.17%	8620	15.40%	83.75%	9701	14.36%	77.20%
**3**	7980	6.46%	86.63%	3424	6.12%	89.86%	4556	6.74%	83.95%
**4**	4754	3.85%	90.48%	1827	3.26%	93.13%	2927	4.33%	88.28%
**5**	3026	2.45%	92.93%	1077	1.92%	95.05%	1949	2.88%	91.16%
**6**	2371	1.92%	94.84%	802	1.43%	96.49%	1569	2.32%	93.49%
**7**	1582	1.28%	96.13%	486	0.87%	97.35%	1096	1.62%	95.11%
**8**	1175	0.95%	97.08%	372	0.66%	98.02%	803	1.19%	96.30%
**9**	908	0.73%	97.81%	273	0.49%	98.51%	635	0.94%	97.24%
**10**	683	0.55%	98.36%	220	0.39%	98.90%	463	0.69%	97.92%
**11**	594	0.48%	98.84%	163	0.29%	99.19%	431	0.64%	98.56%
**12**	422	0.34%	99.19%	126	0.23%	99.42%	296	0.44%	99.00%
**13**	339	0.27%	99.46%	115	0.21%	99.62%	224	0.33%	99.33%
**14**	207	0.17%	99.63%	65	0.12%	99.74%	142	0.21%	99.54%
**15**	168	0.14%	99.76%	48	0.09%	99.82%	120	0.18%	99.72%
**16**	110	0.09%	99.85%	35	0.06%	99.89%	75	0.11%	99.83%
**17**	70	0.06%	99.91%	24	0.04%	99.93%	46	0.07%	99.89%
**18**	54	0.04%	99.95%	16	0.03%	99.96%	38	0.06%	99.95%
**19**	32	0.03%	99.98%	11	0.02%	99.98%	21	0.03%	99.98%
**20**	9	0.01%	99.99%	4	0.01%	99.98%	5	0.01%	99.99%
**21**	2	0.00%	99.99%	1	0.00%	99.99%	1	0.00%	99.99%
**22**	7	0.01%	99.99%	4	0.01%	99.99%	3	0.00%	100.00%
**23**	2	0.00%	100.00%	0	0.00%	100.00%	2	0.00%	100.00%
**24**	0	0.00%	100.00%	0	0.00%	100.00%	0	0.00%	100.00%
**25**	2	0.00%	100.00%	2	0.00%	100.00%	0	0.00%	100.00%
**26**	2	0.00%	100.00%	1	0.00%	100.00%	1	0.00%	100.00%
**29**	1	0.00%	100.00%	1	0.00%	100.00%	0	0.00%	100.00%
	123542	1		55974	100.00%		67568	100.00%	

tab2.5 bis **Statistical parameter of the distributions in tab2.5**

	Min.	1st Qu.	Median	Mean	3rd Qu.	Max.
**all**	1	1	1	2.037	2	29
**male**	1	1	1	1.831	2	29
**female**	1	1	1	2.208	2	26

3-Unique Subjects_2

tab 3.1 **Unique subjects (Number of negative and/or positive outcome per subject)**

# negativ	# positive outcome 0	1	2	3	4	5	6	7	8	9	10	11	12	14
0	0	9933	1828	305	58	13	4	4	3	0	1	0	0	0
1	70788	3635	836	176	59	16	7	5	0	1	1	0	0	0
2	12858	1983	841	339	123	57	18	8	3	1	1	0	0	0
3	4856	805	529	259	106	41	19	6	8	0	2	0	0	0
4	2874	371	320	158	92	33	8	4	2	3	1	0	0	0
5	1715	299	175	107	56	14	12	6	3	0	1	1	0	0
6	1350	200	137	67	32	28	4	5	0	0	1	0	0	0
7	875	159	127	51	29	18	6	1	4	2	0	0	0	0
8	613	124	82	45	27	14	6	6	1	1	1	0	1	0
9	474	107	63	44	11	6	3	1	2	0	1	1	0	1
10	378	73	42	39	11	9	2	1	2	0	0	0	0	0
11	330	69	32	16	8	8	3	0	0	0	0	0	0	0
12	209	43	21	12	9	7	2	0	1	1	0	0	0	0
13	181	29	18	13	4	5	2	0	0	0	0	0	0	0
14	116	22	13	9	7	1	0	0	0	0	0	0	0	0
15	85	9	5	3	3	0	0	0	0	0	0	0	0	0
16	50	5	1	4	0	0	0	0	0	0	0	0	0	0
17	33	6	5	0	0	0	0	0	0	0	0	0	0	0
18	27	1	0	0	1	0	0	0	0	0	0	0	0	0
19	15	1	0	1	1	0	0	0	0	0	0	0	0	0
20	5	0	0	0	0	0	0	0	0	0	0	0	0	0
21	1	1	0	0	0	0	0	0	0	0	0	0	0	0
22	4	0	0	0	0	0	0	0	0	0	0	0	0	0
23	0	0	0	1	0	0	0	0	0	0	0	0	0	0
24	0	1	0	0	0	0	0	0	0	0	0	0	0	0
25	1	0	0	0	0	0	0	0	0	0	0	0	0	0
26	1	0	0	1	0	0	0	0	0	0	0	0	0	0

sum of jellow = subjec with only one tests	80721
sum of sky blue = subjects with more than one positive	15770
sum of green = subjects with more than one negative ou	27051

tab3.2 **Unique subjects (by periods, sex and outcome)**

	I-FMAM	II-JJA	III-SO	IV-ND	
F	24007	12103	16730	14728	67568
	35.53%	17.91%	24.76%	21.80%	######
M	15510	11387	16110	12967	55974
	27.71%	20.34%	28.78%	23.17%	######
tot	39517	23490	32840	27695	123542
	31.99%	19.01%	26.58%	22.42%	######

Negative

	I-FMAM	II-JJA	III-SO	IV-ND	
F	19030	11914	14146	8695	53785
	35.38%	22.15%	26.30%	16.17%	######
M	11509	11182	13660	7703	44054
	26.12%	25.38%	31.01%	17.49%	######
	30539	23096	27806	16398	97840
	31.21%	23.61%	28.42%	16.76%	######

positive

	I-FMAM	II-JJA	III-SO	IV-ND	
F	4977	189	2584	6033	13783
	36.11%	1.37%	18.75%	43.77%	######
M	4001	205	2450	5264	11920
	33.57%	1.72%	20.55%	44.16%	######
	8978	394	5034	11297	25704
	34.93%	1.53%	19.58%	43.95%	######

tab3.3 **Unique subjects (Statistical parameter of age distributions by sex, periods and outcome)**

		Age of ALL subjecs						Age of MALE						Age of FEMALE					
outcome	period	Min.	1st Qu.	Median	Mean	3rd Qu.	Max.	Min.	1st Qu.	Median	Mean	3rd Qu.	Max.	Min.	1st Qu.	Median	Mean	3rd Qu.	Max.
negative	I-FMAM	0	41	54	54.93	69	107	0	39	54	54.33	70	101	0	42	53	55.29	69	107
	II-JJA	0	31	49	49.59	69	106	0	30	49	49.14	69	106	0	31	48	50.02	70	103
	III-SO	0	21	40	40.22	57	104	0	19	37	39.07	57	102	0	23	40	41.33	58	104
	IV-ND	0	29	47	47	63	109	0	28	47	46.11	62	99	0	30	48	47.79	63	109
positive	I-FMAM	0	48	64	63.43	82	108	0	49	63	61.78	78	108	0	48	65	64.76	85	105
	II-JJA	0	25	39	45.54	64	100	0	25	33	40.78	56	97	0	26	47	50.7	77	100
	III-SO	0	28	46	45.79	61	100	0	27	46	45.54	62	99	0	29	46	46.04	60	100
	IV-ND	0	37	53	53.88	72	103	0	36	53	52.59	69	101	0	38	54	55	74	103

Age regardless of outcome

period	Min.	1st Qu.	Median	Mean	3rd Qu.	Max.	
I-FMAM	0	43	55	56.86	74	108	**All**
II-JJA	0	30	49	49.53	69	106	
III-SO	0	22	40	41.07	58	104	
IV-ND	0	32	50	49.81	66	109	
I-FMAM	0	42	56	56.25	73	108	**Male**
II-JJA	0	30	49	48.99	68	106	
III-SO	0	20	39	40.05	58	102	
IV-ND	0	31	49	48.74	65	101	
I-FMAM	0	43	55	57.26	75	107	**Female**
II-JJA	0	31	48	50.03	71	103	
III-SO	0	24	41	42.06	58	104	
IV-ND	0	33	50	50.75	67	109	

tab 3.4 **Unique subjects (Age distributions by period sex and outcome)**

PERIOD: I-FMAM

	all									Male									Female								
	Negative			Positive			all outcome			Negative			Positive			all outcome			Negative			Positive			all outcome		
Age	#	%	cum%	#	%	cum%	#	%	cum%	#	%	cum%	#	%	cum%	#	%	cum%	#	%	cum%	#	%	cum%	#	%	cum%
(0,5]	266	0.9	0.9	27	0.3	0.3	293	0.7	0.7	136	1.2	1.2	14	0.5	0.5	150	1.0	1.0	130	0.7	0.7	13	0.3	0.3	143	0.6	0.6
(5,10]	163	0.5	1.4	26	0.3	0.6	189	0.5	1.2	80	0.7	1.9	15	0.5	0.9	95	0.6	1.6	83	0.4	1.1	11	0.2	0.5	94	0.4	1.0
(10,13]	112	0.4	1.8	19	0.2	0.8	131	0.3	1.6	66	0.6	2.5	6	0.2	1.1	72	0.5	2.0	46	0.2	1.4	13	0.3	0.7	59	0.2	1.2
(13,18]	231	0.8	2.5	60	0.7	1.5	291	0.7	2.3	109	0.9	3.4	28	0.9	2.0	137	0.9	2.9	122	0.6	2.0	32	0.6	1.4	154	0.6	1.9
(18,25]	1119	3.7	6.2	231	2.6	4.0	1350	3.4	5.7	501	4.4	7.8	108	3.5	5.5	609	3.9	6.9	618	3.2	5.2	123	2.5	3.9	741	3.1	5.0
(25,35]	3568	11.7	17.9	664	7.4	11.4	4232	10.7	16.4	1390	12.1	19.8	302	9.8	15.3	1692	10.9	17.8	2178	11.4	16.7	362	7.3	11.1	2540	10.6	15.5
(35,45]	4492	14.7	32.6	822	9.2	20.6	5314	13.4	29.9	1597	13.9	33.7	355	11.5	26.7	1952	12.6	30.3	2895	15.2	31.9	467	9.4	20.5	3362	14.0	29.5
(45,55]	6634	21.7	54.3	1509	16.8	37.4	8143	20.6	50.5	2116	18.4	52.1	651	21.0	47.8	2767	17.8	48.2	4518	23.7	55.6	858	17.2	37.8	5376	22.4	51.9
(55,65]	5281	17.3	71.6	1313	14.6	52.0	6594	16.7	67.2	2008	17.4	69.5	666	21.5	69.3	2674	17.2	65.4	3273	17.2	72.8	647	13.0	50.8	3920	16.3	68.3
(65,75]	2615	8.6	80.2	1080	12.0	64.1	3695	9.4	76.5	1368	11.9	81.4	654	21.1	90.4	2022	13.0	78.5	1247	6.6	79.4	426	8.6	59.3	1673	7.0	75.2
(75,85]	3191	10.4	90.6	1622	18.1	82.1	4813	12.2	88.7	1370	11.9	93.3	788	25.4	115.8	2158	13.9	92.4	1821	9.6	89.0	834	16.8	76.1	2655	11.1	86.3
(85,95]	2565	8.4	99.0	1433	16.0	98.1	3998	10.1	98.8	714	6.2	99.5	391	12.6	128.4	1105	7.1	99.5	1851	9.7	98.7	1042	20.9	97.0	2893	12.1	98.3
(95,110]	302	1.0	100.0	172	1.9	100.0	474	1.2	100.0	54	0.5	100.0	23	0.7	129.2	77	0.5	100.0	248	1.3	100.0	149	3.0	100.0	397	1.7	100.0
tot	30539	100		8978	100		39517	100		11509	100		3097	129		15510	100.0		19030	100.0		4977	100.0		24007	100.0	
%	77.28%			22.72%			######			74.20%			19.97%			100.00%			79.27%			20.73%			100.00%		

PERIOD: II-JJA

	all									Male									Female								
	Negative			Positive			all outcome			Negative			Positive			all outcome			Negative			Positive			all outcome		
Age	#	%	cum%	#	%	cum%	#	%	cum%	#	%	cum%	#	%	cum%	#	%	cum%	#	%	cum%	#	%	cum%	#	%	cum%
(0,5]	539	2.3	2.3	10	2.5	2.5	549	2.3	2.3	270	2.4	2.4	5	2.4	2.4	275	2.4	2.4	269	2.3	2.3	5	2.6	2.6	274	2.3	2.3
(5,10]	459	2.0	4.3	5	1.3	3.8	464	2.0	4.3	241	2.2	4.6	2	1.0	3.4	243	2.1	4.5	218	1.8	4.1	3	1.6	4.2	221	1.8	4.1
(10,13]	328	1.4	5.7	2	0.5	4.3	330	1.4	5.7	173	1.5	6.1	0	0.0	3.4	173	1.5	6.1	155	1.3	5.4	2	1.1	5.3	157	1.3	5.4
(13,18]	709	3.1	8.8	13	3.3	7.6	722	3.1	8.8	395	3.5	9.6	3	1.5	4.9	398	3.5	9.6	314	2.6	8.0	10	5.3	10.6	324	2.7	8.1
(18,25]	1895	8.2	17.0	73	18.5	26.1	1968	8.4	17.2	925	8.3	17.9	48	23.4	28.3	973	8.5	18.1	970	8.1	16.2	25	13.2	23.8	995	8.2	16.3
(25,35]	3482	15.1	32.1	80	20.3	46.4	3562	15.2	32.3	1606	14.4	32.3	56	27.3	55.6	1662	14.6	32.7	1876	15.7	31.9	24	12.7	36.5	1900	15.7	32.0
(35,45]	3008	13.0	45.1	36	9.1	55.6	3044	13.0	45.3	1394	12.5	44.8	16	7.8	63.4	1410	12.4	45.1	1614	13.5	45.5	20	10.6	47.1	1634	13.5	45.5
(45,55]	3355	14.5	59.6	44	11.2	66.8	3399	14.5	59.8	1629	14.6	59.3	22	10.7	74.1	1651	14.5	59.6	1726	14.5	59.9	22	11.6	58.7	1748	14.4	59.9
(55,65]	2707	11.7	71.4	38	9.6	76.4	2745	11.7	71.4	1405	12.6	71.9	21	10.2	84.4	1426	12.5	72.1	1302	10.9	70.9	17	9.0	67.7	1319	10.9	70.8
(65,75]	2341	10.1	81.5	19	4.8	81.2	2360	10.0	81.5	1256	11.2	83.1	8	3.9	88.3	1264	11.1	83.2	1085	9.1	80.0	11	5.8	73.5	1096	9.1	79.9
(75,85]	2792	12.1	93.6	42	10.7	91.9	2834	12.1	93.6	1349	12.1	95.2	19	9.3	97.6	1368	12.0	95.2	1443	12.1	92.1	23	12.2	85.7	1466	12.1	92.0
(85,95]	1387	6.0	99.6	24	6.1	98.0	1411	6.0	99.6	515	4.6	99.8	4	2.0	99.5	519	4.6	99.8	872	7.3	99.4	20	10.6	96.3	892	7.4	99.4
(95,110]	94	0.4	100.0	8	2.0	100.0	102	0.4	100.0	24	0.2	100.0	1	0.5	100.0	25	0.2	100.0	70	0.6	100.0	7	3.7	100.0	77	0.6	100.0
tot	23096	100		394	100		23490	100		11182	100		205	100		11387	100.0		11914	100.0		189	100.0		12103	100.0	
%	98.32%			1.68%			######			98.20%			1.80%			100.00%			98.44%			1.56%			100.00%		

tab 3.4 continue

PERIOD: III-SO

	tutti									Male									Female								
	Negative			Positive			all outcome			Negative			Positive			all outcome			Negative			Positive			all outcome		
Age	#	%	cum%	#	%	cum%	#	%	cum%	#	%	cum%	#	%	cum%	#	%	cum%	#	%	cum%	#	%	cum%	#	%	cum%
(0,5]	1476	5.3	5.3	98	1.9	1.9	1574	4.8	4.8	813	6.0	6.0	48	2.0	2.0	861	5.3	5.3	663	4.7	4.7	50	1.9	1.9	713	4.3	4.3
(5,10]	1563	5.6	10.9	123	2.4	4.4	1686	5.1	9.9	800	5.9	11.8	59	2.4	4.4	859	5.3	10.7	763	5.4	10.1	64	2.5	4.4	827	4.9	9.2
(10,13]	1213	4.4	15.3	151	3.0	7.4	1364	4.2	14.1	677	5.0	16.8	81	3.3	7.7	758	4.7	15.4	536	3.8	13.9	70	2.7	7.1	606	3.6	12.8
(13,18]	1963	7.1	22.4	280	5.6	13.0	2243	6.8	20.9	1110	8.1	24.9	172	7.0	14.7	1282	8.0	23.3	853	6.0	19.9	108	4.2	11.3	961	5.7	18.6
(18,25]	2491	9.0	31.3	412	8.2	21.1	2903	8.8	29.8	1246	9.1	34.0	193	7.9	22.6	1439	8.9	32.3	1245	8.8	28.7	219	8.5	19.8	1464	8.8	27.3
(25,35]	4127	14.8	46.2	722	14.3	35.5	4849	14.8	44.5	1951	14.3	48.3	347	14.2	36.7	2298	14.3	46.5	2176	15.4	44.1	375	14.5	34.3	2551	15.2	42.6
(35,45]	3525	12.7	58.8	684	13.6	49.1	4209	12.8	57.3	1663	12.2	60.5	291	11.9	48.6	1954	12.1	58.7	1862	13.2	57.2	393	15.2	49.5	2255	13.5	56.0
(45,55]	3876	13.9	72.8	917	18.2	67.3	4793	14.6	71.9	1775	13.0	73.5	437	17.8	66.4	2212	13.7	72.4	2101	14.9	72.1	480	18.6	68.1	2581	15.4	71.5
(55,65]	2991	10.8	83.5	625	12.4	79.7	3616	11.0	82.9	1436	10.5	84.0	298	12.2	78.6	1734	10.8	83.2	1555	11.0	83.1	327	12.7	80.7	1882	11.2	82.7
(65,75]	1976	7.1	90.6	428	8.5	88.2	2404	7.3	90.3	1044	7.6	91.6	247	10.1	88.7	1291	8.0	91.2	932	6.6	89.7	181	7.0	87.7	1113	6.7	89.4
(75,85]	1797	6.5	97.1	355	7.1	95.3	2152	6.6	96.8	821	6.0	97.6	180	7.3	96.0	1001	6.2	97.4	976	6.9	96.6	175	6.8	94.5	1151	6.9	96.3
(85,95]	754	2.7	99.8	221	4.4	99.6	975	3.0	99.8	306	2.2	99.9	94	3.8	99.9	400	2.5	99.9	448	3.2	99.7	127	4.9	99.4	575	3.4	99.7
(95,110]	54	0.2	100.0	18	0.4	100.0	72	0.2	100.0	18	0.1	100.0	3	0.1	100.0	21	0.1	100.0	36	0.3	100.0	15	0.6	100.0	51	0.3	100.0
	27806	100		5034	100		32840	100		13660	100		2450	100		16110	100.0		14146	100.0		2584	100.0		16730	100.0	
%	84.67%			15.33%			######			84.79%			15.21%			100.00%			84.55%			15.45%			100.00%		

PERIOD: IV-ND

	all									Male									Female								
	Negative			Positive			all outcome			Negative			Positive			all outcome			Negative			Positive			all outcome		
Age	#	%	cum%	#	%	cum%	#	%	cum%	#	%	cum%	#	%	cum%	#	%	cum%	#	%	cum%	#	%	cum%	#	%	cum%
(0,5]	311	1.9	1.9	64	0.6	0.6	375	1.4	1.4	168	2.2	2.2	31	0.6	0.6	199	1.5	1.5	143	1.6	1.6	33	0.5	0.5	176	1.2	1.2
(5,10]	337	2.1	4.0	81	0.7	1.3	418	1.5	2.9	187	2.4	4.6	45	0.9	1.4	232	1.8	3.3	150	1.7	3.4	36	0.6	1.1	186	1.3	2.5
(10,13]	253	1.5	5.5	104	0.9	2.2	357	1.3	4.2	142	1.8	6.5	51	1.0	2.4	193	1.5	4.8	111	1.3	4.6	53	0.9	2.0	164	1.1	3.6
(13,18]	717	4.4	9.9	262	2.3	4.5	979	3.5	7.7	393	5.1	11.6	134	2.5	5.0	527	4.1	8.9	324	3.7	8.4	128	2.1	4.1	452	3.1	6.6
(18,25]	1527	9.3	19.2	738	6.5	11.1	2265	8.2	15.9	718	9.3	20.9	356	6.8	11.7	1074	8.3	17.2	809	9.3	17.7	382	6.3	10.5	1191	8.1	14.7
(25,35]	2447	14.9	34.1	1365	12.1	23.1	3812	13.8	29.6	1124	14.6	35.5	661	12.6	24.3	1785	13.8	30.9	1323	15.2	32.9	704	11.7	22.1	2027	13.8	28.5
(35,45]	2161	13.2	47.3	1470	13.0	36.2	3631	13.1	42.7	977	12.7	48.2	686	13.0	37.3	1663	12.8	43.7	1184	13.6	46.5	784	13.0	35.1	1968	13.4	41.9
(45,55]	2760	16.8	64.1	2018	17.9	54.0	4778	17.3	60.0	1267	16.4	64.6	905	17.2	54.5	2172	16.8	60.5	1493	17.2	63.7	1113	18.4	53.6	2606	17.7	59.5
(55,65]	2345	14.3	78.4	1656	14.7	68.7	4001	14.4	74.4	1107	14.4	79.0	829	15.7	70.3	1936	14.9	75.4	1238	14.2	77.9	827	13.7	67.3	2065	14.0	73.6
(65,75]	1552	9.5	87.9	1190	10.5	79.2	2742	9.9	84.3	786	10.2	89.2	641	12.2	82.4	1427	11.0	86.4	766	8.8	86.7	549	9.1	76.4	1315	8.9	82.5
(75,85]	1311	8.0	95.9	1359	12.0	91.2	2670	9.6	94.0	599	7.8	96.9	639	12.1	94.6	1238	9.5	96.0	712	8.2	94.9	720	11.9	88.3	1432	9.7	92.2
(85,95]	619	3.8	99.6	906	8.0	99.3	1525	5.5	99.5	221	2.9	99.8	273	5.2	99.8	494	3.8	99.8	398	4.6	99.5	633	10.5	98.8	1031	7.0	99.2
(95,110]	58	0.4	100.0	84	0.7	100.0	142	0.5	100.0	14	0.2	100.0	13	0.2	100.0	27	0.2	100.0	44	0.5	100.0	71	1.2	100.0	115	0.8	100.0
tot	16398	100		11297	100		27695	100		7703	100		5264	100		12967	100.0		8695	100.0		6033	100.0		14728	100.0	
%	59.21%			40.79%			######			59.40%			40.60%			100.00%			59.04%			40.96%			100.00%		

4-Health. Work.-S symth. cat

unigrams for healthcare workers: oper, operarator, operator, operatore, operatric, opperator, sanit, sanita, sanitariao, senitario, oss, infermier,

tab4.1 **Healthcare workers and test otucome**

Outcome	Others	Healt. Work.	tot	%	% Other	% Healt. Work.
NEGATIVE	127723	40276	167999	85.29%	82.96%	93.62%
POSITIVE	26227	2744	28971	14.71%	17.04%	6.38%
tot	153950	43020	196970		100.00%	100.00%
	78.16%	21.84%	100.00%			

tab4.2 **Healthcare workers and periods**

Period	Others	Healt. Work.	tot	% Healt. Work. in period
I-FMAM	48170	22216	70386	31.56%
II-JJA	34019	4568	38587	11.84%
III-SO	44735	13744	58479	23.50%
IV-ND	27026	2492	29518	8.44%
tot esito	153950	43020	196970	
%tot esito	78.16%	21.84%		

tab4.3 **Healthcare workers and test outcome by period**

		NEGATIVE	POSITIVE	
I-FMAM	Healt. Work	20277	1939	22216
		91.27%	8.73%	100%
	Others	36781	11389	48170
		76.36%	23.64%	100%
II-JJA	Healt. Work	4513	55	4568
		98.80%	1.20%	100%
	Others	32784	1235	34019
		96.37%	3.63%	100%
III-SO	Healt. Work	13326	418	13744
		96.96%	3.04%	100.00%
	Others	39833	4902	44735
		89.04%	10.96%	100.00%
IV-ND	Healt. Work	2160	332	2492
		86.68%	13.32%	100%
	Others	18325	8701	27026
		67.81%	32.19%	100%

tab4.4 Healthcare workers- and COVID-19 related symptoms

	Others	Healt. Work.	tot	%	Other	Healt. Work.
**Asymptomatic**	126481	41706	168187	85.39%	82.16%	96.95%
**Symptomatic**	27469	1314	28783	14.61%	17.84%	3.05%
tot	153950	43020	196970		100.00%	100.00%
	78.16%	21.84%	100.00%			

tab4.5 Only sympomatic records: Healthcare workers- and COVID-19 related symptoms

	Others	Healt. Work.	tot		Other	Healt. Work.
**NEGATIVE**	21129	1032	22161	76.99%	76.92%	78.54%
**POSITIVE**	6340	282	6622	23.01%	23.08%	21.46%
tot	27469	1314	28783		100.00%	100.00%
	95.43%	4.57%	100.00%			

tab4.6 Healthcare workers- and COVID-19 related symptoms by period

		Asymptomatic	Simptomatic	
I-FMAM	**Healt. Work**	21303	913	22216
		95.89%	4.11%	100%
	**Others**	34321	13849	48170
		71.25%	28.75%	100%
II-GLA	**Healt. Work**	4446	122	4568
		97.33%	2.67%	100%
	**Others**	29360	4659	34019
		86.30%	13.70%	100%
III-SO	**Healt. Work**	13487	257	13744
		98.13%	1.87%	######
	**Others**	39242	5493	44735
		87.72%	12.28%	######
IV-ND	**Healt. Work**	2470	22	2492
		99.12%	0.88%	100%
	**Others**	23558	3468	27026
		87.17%	12.83%	100%

5-Ass.homes guests-S symth. cat

unigrams for assistence homes guests: cas, casa, comunit, comunita, dimora, resident, residenz, residenzial, residenzialit, residenzialt dormitorioi, fissa, ospit, ospite, resid, riposo, rra, rsa, struttura,vagabondaggi

tab5.1 Ass. Homes guest and test outcome

	Others	Guests	tot	%	% Others	% Guest
NEGATIVE	105460	22263	127723	82.96%	82.89%	83.34%
POSITIVE	21775	4452	26227	17.04%	17.11%	16.66%
tot	127235	26715	153950		100.00%	100.00%
	82.65%	17.35%	100.00%			

tab5.2 Ass. Homes guests and period

	Others	Guests	tot period	% Guest in period
I-FMAM	36931	11239	48170	23.33%
II-GLA	27831	6188	34019	18.19%
III-SO	38668	6067	44735	13.56%
IV-ND	23805	3221	27026	11.92%
tot esito	127235	26715	153950	
%tot esito	82.65%	17.35%		

tab5.3 Ass. Homes guests and test outcome by period

		NEGATIVE	POSITIVE	
I-FMAM	Guests	8238	3001	11239
		73.30%	26.70%	100%
	Others	28543	8388	36931
		77.29%	22.71%	100%
II-JJA	Guests	5873	315	6188
		94.91%	5.09%	100%
	Others	26911	920	27831
		96.69%	3.31%	100%
III-SO	Guests	5757	310	6067
		94.89%	5.11%	100.00%
	Others	34076	4592	38668
		88.12%	11.88%	100.00%
IV-ND	Guests	2395	826	3221
		74.36%	25.64%	100%
	Others	15930	7875	23805
		66.92%	33.08%	100%

tab5.4 Ass. Homes guests and COVID-19 related symptoms

	Others	Guests	tot	%	Others	Guests
**Asymptomatic**	100751	25730	126481	82.16%	79.18%	96.31%
**Symptomatic**	26484	985	27469	17.84%	20.82%	3.69%
tot	127235	26715	153950		100.00%	100.00%
	82.65%	17.35%	100.00%			

tab5.5 Only sympomatic records: Ass. Homes guests- and COVID-19 related symptoms

	Others	Guests	tot		0	1
**NEGATIVE**	20540	589	21129	76.92%	77.56%	59.80%
**POSITIVE**	5944	396	6340	23.08%	22.44%	40.20%
Sum	26484	985	27469		100.00%	100.00%
	96.41%	3.59%	100.00%			

tab5.6 Ass. Homes guests and COVID-19 related symptoms by period

		Asymptomatic	Simptomatic	
I-FMAM	**Guests**	10487	752	11239
		93.31%	6.69%	100%
	**Others**	23834	13097	36931
		64.54%	35.46%	100%
II-JJA	**Guests**	6085	103	6188
		98.34%	1.66%	100%
	**Others**	23275	4556	27831
		83.63%	16.37%	100%
III-SO	**Guests**	5950	117	6067
		98.07%	1.93%	100.00%
	**Others**	33292	5376	38668
		86.10%	13.90%	100.00%
IV-ND	**Guests**	3208	13	3221
		99.60%	0.40%	100%
	**Others**	20350	3455	23805
		85.49%	14.51%	100%

6-positive contacts subj-S symt

unigrams for positive contacts subject: contatoi, contattio, contatto, positi, positiv, positivit, positivo

tab6.1 Contacts with positive and test outcome

	Others	Cont. Pos.	tot	%	Others	Cont. Pos.
**NEGATIVE**	102102	25621	127723	82.96%	84.84%	76.25%
**POSITIVE**	18245	7982	26227	17.04%	15.16%	23.75%
tot	120347	33603	153950		100.00%	100.00%
	78.17%	21.83%	100.00%			

tab6.2 Contacts with positive and period

	Others	Cont. Pos.	tot period	% Cont. Pos. In period
I-FMAM	35963	12207	48170	25.34%
II-JJA	31494	2525	34019	7.42%
III-SO	37186	7549	44735	16.87%
IV-ND	15704	11322	27026	41.89%
tot esito	127235	26715	153950	
%tot esito	82.65%	17.35%		

tab6.3 Contacts with positive and test outcome by period

		NEGATIVO	POSITIVO	
I-FMAM	Cont. Pos.	8960	3247	12207
		73.40%	26.60%	100%
	Others	27821	8142	35963
		77.36%	22.64%	100%
II-JJA	Cont. Pos.	2394	131	2525
		94.81%	5.19%	100%
	Others	30390	1104	31494
		96.49%	3.51%	100%
III-SO	Cont. Pos.	6436	1113	7549
		85.26%	14.74%	100.00%
	Others	33397	3789	37186
		89.81%	10.19%	100.00%
IV-ND	Cont. Pos.	7831	3491	11322
		69.17%	30.83%	100%
	Others	10494	5210	15704
		66.82%	33.18%	100%

tab6.4 Contacts with positive and COVID-19 related symptoms

	Others	Cont. Pos.	tot	%	Others	Cont. Pos.
**Asymptomatic**	99006	27475	126481	82.16%	82.27%	81.76%
**Symptomatic**	21341	6128	27469	17.84%	17.73%	18.24%
tot	120347	33603	153950		100.00%	100.00%
	78.17%	21.83%	100.00%			

tab6.5 Only sympomatic records:Contacts with positive and COVID-19 related symptoms

	Others	Cont. Pos.	tot	%	Others	Cont. Pos.
**NEGATIVE**	16796	4333	21129	76.92%	78.70%	70.71%
**POSITIVE**	4545	1795	6340	23.08%	21.30%	29.29%
tot	21341	6128	27469		100.00%	100.00%
	77.69%	22.31%	100.00%			

tab6.6 Contacts with positive and COVID-19 related symptoms by period

		Asymptomatic	Simptomatic	
I-FMAM	**Cont. Pos.**	8958	3249	12207
		73.38%	26.62%	100%
	**Others**	25363	10600	35963
		70.53%	29.47%	100%
II-JJA	**Cont. Pos.**	2441	84	2525
		96.67%	3.33%	100%
	**Others**	26919	4575	31494
		85.47%	14.53%	100%
III-SO	**Cont. Pos.**	7124	425	7549
		94.37%	5.63%	100.00%
	**Others**	32118	5068	37186
		86.37%	13.63%	100.00%
IV-ND	**Cont. Pos.**	8952	2370	11322
		79.07%	20.93%	100%
	**Others**	14606	1098	15704
		93.01%	6.99%	100%

7-COVID-19 and Pathologies sympthoms

tab7.1 COVID-19 related symptoms and test outcome

	Asymptomatic	Simptomatic	tot	%	Asymptomatic	Simptomatic
**NEGATIV**	145838	22161	167999	85.29%	86.71%	76.99%
**POSITIVE**	22349	6622	28971	14.71%	13.29%	23.01%
tot	168187	28783	196970		100.00%	100.00%
	85.39%	14.61%	100.00%			

tab7.2 Others Pathologies related symtoms and test outcome

	No Pathologies	Pathologies	tot	%	No Pathologies	Pathologies
**NEGATIV**	156948	11051	167999	85.29%	84.93%	90.74%
**POSITIVE**	27843	1128	28971	14.71%	15.07%	9.26%
tot	184791	12179	196970		100.00%	100.00%
	93.82%	6.18%	100.00%			

tab7.3 COVID-19 related symptoms and test outcome by period

		NEGATIVE	POSITIVE	
I-FMAM	**Symptomatic**	10287	4475	14762
		69.69%	30.31%	100%
	**Asymptomatic**	46771	8853	55624
		84.08%	15.92%	100%
II-JJA	**Symptomatic**	4726	55	4781
		98.85%	1.15%	100%
	**Asymptomatic**	32571	1235	33806
		96.35%	3.65%	100%
III-SO	**Symptomatic**	4546	1204	5750
		79.06%	20.94%	100.00%
	**Asymptomatic**	48613	4116	52729
		92.19%	7.81%	100.00%
IV-ND	**Symptomatic**	2602	888	3490
		74.56%	25.44%	100%
	**Asymptomatic**	17883	8145	26028
		68.71%	31.29%	100%

tab7.4 COVID-19 related symptoms and test outcome by sex

	MALE						FEMALE						ALL					
	Asymptomatic		Simptomatic		tot		Asymptomatic		Simptomatic		tot		Asymptomatic		Simptomatic		tot	
		%		%		%		%		%		%		%		%		%
NEGATIVE	57400	85.6%	10451	74.8%	67851	83.8%	88438	87.4%	11710	79.1%	100148	86.4%	145838	86.7%	22161	77.0%	167999	85.3%
POSITIVE	9620	14.4%	3527	25.2%	13147	16.2%	12729	12.6%	3095	20.9%	15824	13.6%	22349	13.3%	6622	23.0%	28971	14.7%
tot	67020	100.0%	13978	100.0%	80998	100.0%	101167	100.0%	14805	100.0%	115972	100.0%	168187	100.0%	28783	100.0%	196970	100.0%
%	82.7%		17.3%		100.0%		87.2%		12.8%		100.0%		85.4%		14.6%		100.0%	

tab7.5 **Only Sympomatic Records:** Age by outcome and sex

	all									Male									Female								
	Negative			Positive			tot			Negative			Positive			tot			Negative			Positive			tot		
**Age**	#	%	cum%	#	%	cum%	#	%	cum%	#	%	cum%	#	%	cum%	#	%	cum%	#	%	cum%	#	%	cum%	#	%	cum%
(0,5]	853	3.8	3.8	37	0.6	0.6	890	3.1	3.1	455	4.4	4.4	20	0.6	0.6	475	3.4	3.4	398	3.4	3.4	17	0.5	0.5	415	2.8	2.8
(5,10]	443	2.0	5.8	42	0.6	1.2	485	1.7	4.8	216	2.1	6.4	26	0.7	1.3	242	1.7	5.1	227	1.9	5.3	16	0.5	1.1	243	1.6	4.4
(10,13]	257	1.2	7.0	43	0.6	1.8	300	1.0	5.8	134	1.3	7.7	19	0.5	1.8	153	1.1	6.2	123	1.1	6.4	24	0.8	1.8	147	1.0	5.4
(13,18]	563	2.5	9.5	103	1.6	3.4	666	2.3	8.1	319	3.1	10.8	56	1.6	3.4	375	2.7	8.9	244	2.1	8.5	47	1.5	3.4	291	2.0	7.4
(18,25]	1169	5.3	14.8	249	3.8	7.2	1418	4.9	13.1	549	5.3	16.0	130	3.7	7.1	679	4.9	13.8	620	5.3	13.8	119	3.8	7.2	739	5.0	12.4
(25,35]	2291	10.3	25.2	561	8.5	15.6	2852	9.9	23.0	981	9.4	25.4	293	8.3	15.4	1274	9.1	22.9	1310	11.2	25.0	268	8.7	15.9	1578	10.7	23.1
(35,45]	2562	11.6	36.7	620	9.4	25.0	3182	11.1	34.0	1129	10.8	36.2	288	8.2	23.6	1417	10.1	33.0	1433	12.2	37.2	332	10.7	26.6	1765	11.9	35.0
(45,55]	3139	14.2	50.9	1158	17.5	42.5	4297	14.9	49.0	1426	13.6	49.8	610	17.3	40.9	2036	14.6	47.6	1713	14.6	51.8	548	17.7	44.3	2261	15.3	50.2
(55,65]	2840	12.8	63.7	1094	16.5	59.0	3934	13.7	62.6	1361	13.0	62.9	614	17.4	58.3	1975	14.1	61.7	1479	12.6	64.4	480	15.5	59.8	1959	13.2	63.5
(65,75]	2692	12.1	75.8	967	14.6	73.6	3659	12.7	75.3	1425	13.6	76.5	592	16.8	75.1	2017	14.4	76.1	1267	10.8	75.3	375	12.1	71.9	1642	11.1	74.6
(75,85]	3387	15.3	91.1	1120	16.9	90.5	4507	15.7	91.0	1674	16.0	92.5	614	17.4	92.5	2288	16.4	92.5	1713	14.6	89.9	506	16.3	88.3	2219	15.0	89.6
(85,95]	1847	8.3	99.5	593	9.0	99.5	2440	8.5	99.5	747	7.1	99.7	252	7.1	99.6	999	7.1	99.7	1100	9.4	99.3	341	11.0	99.3	1441	9.7	99.3
(95,110]	118	0.5	100.0	35	0.5	100.0	153	0.5	100.0	35	0.3	100.0	13	0.4	100.0	48	0.3	100.0	83	0.7	100.0	22	0.7	100.0	105	0.7	100.0
tot	22161	100		6622	100		28783	100		10451	100		3527	100		13978	100.0		11710	100.0		3095	100.0		14805	100.0	
%	76.99%			23.01%			100.0%			74.77%			25.23%			100.0%			79.09%			20.91%			100.0%		

tab7.6 **Only Asympomatic Records:** Age by outcome and sex

	all									Male									Female								
	Negative			Positive			tot			Negative			Positive			tot			Negative			Positive			tot		
**Age**	#	%	cum%	#	%	cum%	#	%	cum%	#	%	cum%	#	%	cum%	#	%	cum%	#	%	cum%	#	%	cum%	#	%	cum%
(0,5]	1936	1.3	1.3	174	0.8	0.8	2110	1.3	1.3	1014	1.8	1.8	83	0.9	0.9	1097	1.6	1.6	922	1.0	1.0	91	0.7	0.7	1013	1.0	1.0
(5,10]	2307	1.6	2.9	214	1.0	1.7	2521	1.5	2.8	1183	2.1	3.8	108	1.1	2.0	1291	9.2	10.9	1124	1.3	2.3	106	0.8	1.5	1230	1.2	2.2
(10,13]	1883	1.3	4.2	274	1.2	3.0	2157	1.3	4.0	1037	1.8	5.6	134	1.4	3.4	1171	8.4	19.3	846	1.0	3.3	140	1.1	2.6	986	1.0	3.2
(13,18]	3547	2.4	6.6	579	2.6	5.6	4126	2.5	6.5	1945	3.4	9.0	314	3.3	6.6	2259	16.2	35.4	1602	1.8	5.1	265	2.1	4.7	1867	1.8	5.0
(18,25]	8221	5.6	12.3	1422	6.4	11.9	9643	5.7	12.2	3750	6.5	15.6	698	7.3	13.9	4448	31.8	67.2	4471	5.1	10.1	724	5.7	10.4	5195	5.1	10.2
(25,35]	20011	13.7	26.0	2692	12.0	24.0	22703	13.5	25.7	8425	14.7	30.2	1301	13.5	27.4	9726	69.6	136.8	11586	13.1	23.2	1391	10.9	21.3	12977	12.8	23.0
(35,45]	20995	14.4	40.4	2643	11.8	35.8	23638	14.1	39.8	7779	13.6	43.8	1136	11.8	39.2	8915	63.8	200.6	13216	14.9	38.2	1507	11.8	33.2	14723	14.6	37.6
(45,55]	29140	20.0	60.4	3861	17.3	53.1	33001	19.6	59.4	9658	16.8	60.6	1632	17.0	56.2	11290	80.8	281.4	19482	22.0	60.2	2229	17.5	50.7	21711	21.5	59.0
(55,65]	21027	14.4	74.8	2893	12.9	66.0	23920	14.2	73.6	8314	14.5	75.1	1379	14.3	70.5	9693	69.3	350.7	12713	14.4	74.6	1514	11.9	62.6	14227	14.1	73.1
(65,75]	10666	7.3	82.1	1935	8.7	74.7	12601	7.5	81.1	5665	9.9	85.0	1049	10.9	81.4	6714	48.0	398.7	5001	5.7	80.2	886	7.0	69.5	5887	5.8	78.9
(75,85]	13869	9.5	91.6	2803	12.5	87.2	16672	9.9	91.0	5662	9.9	94.8	1173	12.2	93.6	6835	48.9	447.6	8207	9.3	89.5	1630	12.8	82.4	9837	9.7	88.6
(85,95]	11009	7.5	99.2	2547	11.4	98.6	13556	8.1	99.1	2804	4.9	99.7	584	6.1	99.7	3388	24.2	471.9	8205	9.3	98.8	1963	15.4	97.8	10168	10.1	98.7
(95,110]	1227	0.8	100.0	312	1.4	100.0	1539	0.9	100.0	164	0.3	100.0	29	0.3	100.0	193	1.4	473.3	1063	1.2	100.0	283	2.2	100.0	1346	1.3	100.0
tot	145838	100		22349	100		168187	100		57400	100		9620	100		67020	473.3		88438	100.0		12729	100.0		101167	100.0	
%	86.71%			13.29%			100.0%			85.65%			14.35%			100.0%			87.42%			12.58%			100.0%		
